# Enhanced immunity after Ad26.COV2.S vaccine breakthrough infection

**DOI:** 10.1016/j.xcrm.2022.100579

**Published:** 2022-03-15

**Authors:** David C. Montefiori

**Affiliations:** 1 Department of Surgery, Duke University Medical Center, Durham, NC, USA

## Abstract

A study by Kitchin, Richardson et al. finds that breakthrough infection with the SARS-CoV-2 Delta variant in Ad26.COV2.S-vaccinated individuals induces strong neutralizing antibody responses against multiple variants, including the Omicron variant.

## Main text

Potent and durable immunity against current and future variants of SARS-CoV-2 in large segments of the world’s population is essential to the control of the COVD-19 pandemic. Immunity can be achieved through prior infection (natural immunity), vaccination, or a combination of both. Notably, the natural immunity that is acquired after recovery from SARS-CoV-2 infection can set the stage for unexpectedly potent immune responses after a single dose of the Pfizer-BioNTech BNT162b2 or Moderna mRNA-1273 vaccines,^1^, ^2^, ^3^ and also after a single dose of the Janssen (Johnson & Johnson) Ad26.COV2.S non-replicating viral vector vaccine.^4^ One hallmark of this enhanced immunity is the production of high titer neutralizing antibodies against multiple SARS-CoV-2 variants, which has received a lot of attention due to the recognition that neutralizing antibodies are a key biomarker for COVID-19 vaccines.^5^ Underlying these remarkable responses are increases in memory B cells and improved help from CD4^+^ T cells and T_FH_ cells, all contributing to a type of “hybrid immunity” orchestrated by a combination of natural infection and vaccination.^6^

Enhanced immunity also appears to be a feature of vaccine breakthrough infections. In one study, high levels of neutralizing antibodies were detected against ancestral WA-1 and the Alpha, Beta, and Delta variants of SARS-CoV-2 that exceeded the levels induced by vaccination alone.^7^ Notably, the levels of neutralizing antibodies after vaccine breakthrough infection were similar to those seen after vaccination in the setting of prior infection. In another study, the levels of neutralizing antibodies against the D614G, Delta, Beta, and Omicron variants were much higher after breakthrough infection than after two doses of mRNA vaccine alone and were similar to levels seen after a third dose of mRNA vaccine.^8^

Neutralizing antibodies are not the only functional immune response to be augmented after vaccine breakthrough infection, or after vaccination in the setting of prior infection. Equally elevated levels of antibody-dependent cellular phagocytosis (ADCP) activity were seen in people infected before or after vaccination compared to people vaccinated in the absence of infection.^7^ Other studies detected robust antibody-dependent cellular cytotoxicity (ADCC) activity against multiple variants in previously infected individuals after a single dose of either an mRNA vaccine^9^ or Ad26.COV2.S vaccine.^4^

The heightened immunity and vaccine dose-sparing effect achieved through a combination of infection and vaccination (in either order) has important implications for control of the COVID-19 pandemic, especially considering the increased numbers of vaccine breakthrough cases occurring with the highly contagious Omicron variant. Studies of immunity in breakthrough cases have mainly focused on mRNA vaccines, with little attention to the Ad26.COV2.S vaccine that has been widely distributed in many countries as a single dose vaccine. The Ad26.COV2.S vaccine is known to elicit relatively low levels of neutralizing antibodies and to have modestly reduced efficacy compared to mRNA vaccines. Thus, the impact of breakthrough infection on subsequent immunity in this vaccinated population is of high importance.

A study by Kitchin, Richardson et al.^10^ begins to provide answers to what this impact might be. They measured plasma antibodies in 19 Ad26.COV2.S-vaccinated health care workers (HCWs) in South Africa, 6 of whom experienced mild cases of breakthrough infections 4–5 months later, during a period of time when Delta was the predominant variant in the region. Neutralizing antibodies were assessed with pseudoviruses expressing the spike proteins of prototypic SARS-CoV-2 (Wuhan-1 containing a single D614G spike mutation, hereafter referred to as the D614G variant), three variants of concern (Beta, Delta, and Omicron), and SARS-CoV-1.

Neutralizing antibodies prior to infection were low or undetectable against all variants, especially against Omicron and SARS-CoV-1, for which results were mostly negative. Within 1 month after breakthrough infection, high titers of neutralizing antibodies were detected against all of the SARS-CoV-2 variants in at least 5/6 cases examined. Moreover, low to moderate titers were detected against the more distant SARS-CoV-1 in all but one case. For the most part, the neutralizing titers against the D614G variant exceeded the geometric mean titer elicited by a 2-dose regimen of the Pfizer-BioNTech BNT162b2 vaccine, and they also exceeded the titers in convalescent plasma from people who experienced either moderate or severe COVID-19. Moreover, breakthrough infection in this study was associated with robust ADCC activity that was cross-reactive against the D614G, Beta, and Delta variants.

Based on these results, a single dose of the Ad26.COV2.S vaccine appears capable of priming for potent humoral immunity across multiple SARS-CoV-2 variants after recovery from subsequent SARS-CoV-2 infection, much the same as mRNA vaccines. These new findings suggest that immunity acquired through a combination of SARS-CoV-2 infection and Ad26.COV2.S vaccination could have a positive impact on the COVID-19 pandemic in regions where this vaccine is deployed and vaccine boosters are either not readily available or their uptake is low.

The main limitations of this study are the small number of breakthrough cases examined, and the fact that all cases are likely from infection with the Delta variant. It will be informative to examine additional cases, especially breakthrough infections with other variants, for their potential to differentially influence the levels of cross-immunity.^4^ It will also be important to assess whether vaccine breakthrough infection improves the durability of immune responses compared to infection or vaccination alone.
